# The positive rates of hepatitis B surface antibody in youth after booster vaccination: a 4-year follow-up study with large sample

**DOI:** 10.1042/BSR20210182

**Published:** 2021-09-20

**Authors:** Xia Zhu, Juan Wang, Ming Wang, Ling-yao Du, Yu-lin Ji, Xuan Zhang, Hong Tang

**Affiliations:** 1 Center of Infectious Diseases, West China Hospital of Sichuan University, No.37 Guo Xue Xiang, Chengdu 610041, P.R. China; 2 Respiratory and Critical Care Medicine Department, West China Hospital of Sichuan University, Chengdu 610041, P.R. China

**Keywords:** anti-HBs, Booster vaccination, positive rates, youths

## Abstract

**Background:** Hepatitis B virus (HBV) infection is still a public issue in the world. Hepatitis B vaccination is widely used as an effective measure to prevent HBV infection. This large-sample study aimed to evaluate the positive rates of hepatitis B surface antibody (anti-HBs) in youth after booster vaccination.

**Methods:** A total of 37788 participants were divided into two groups according to the baseline levels of anti-HBs before booster vaccination: the negative group (anti-HBs(−)) and the positive group (anti-HBs(+)). Participants were tested for anti-HBs levels after receiving a booster vaccine at 1 and 4 years.

**Results:** The positive rates of anti-HBs were 34.50%, 73.80% and 67.32% before booster vaccination at 1 and 4 years after vaccination, respectively. At 4 years after the booster vaccination, the positive rates of 13–18 years were 47.54%, which was the lowest level among all youth age groups. In the anti-HBs(−) group, the positive conversion rates of anti-HBs were 74.62% at 1 year after receiving a booster vaccine, and 67.66% at 4 years after vaccination. In the anti-HBs(+) group, the positive maintenance rates of anti-HBs were 70.16% after 1 year, and 66.66% after 4 years. Compared with the baseline anti-HBs (+) group, the positive rates of the baseline anti-HBs(−) group were higher at 1 and 4 years after receiving the booster vaccine.

**Conclusion:** The positive rates of anti-HBs declined over time, especially the positive maintenance rates were the lowest at age of 13–18 years.

## Introduction

Hepatitis B virus (HBV) infection can cause both acute and chronic liver diseases [1,2]. According to World Health Organization (WHO) report, more than 250 million people were infected with HBV and resulted in 887000 deaths in 2015 [3]. China is one of the highly endemic areas of HBV infection, with an estimated 93 million carriers of HBV [4]. At present, there is no long-term effective treatment for HBV infection and infection-related diseases. Hepatitis B vaccination is a safe and effective measure that can reduce the risk of HBV-related complications [5]. The 2014 Chinese serosurvey report showed that the HBV infection rates of the population aged 1–29 years have been significantly reduced after decades of hepatitis B vaccination nationwide, especially for children under 5 years decreased by 97% [6].

The primary immunization following the vaccination schedule after birth may not be enough to protect youth from HBV infection, because the protective antibodies induced by the hepatitis B vaccine will gradually disappear over time [7–9]. It is widely considered that hepatitis B surface antibody (anti-HBs) exceeding 10 mIU/ml has a serum protective effect, the risk of infection increases when antibody titers are less than 10 mIU/ml [10,11]. Previous research has reported that approximately 5% of HBV vaccine recipients may still be infected with HBV [12,13]. Some researchers suggest that the use of booster dose vaccine may be necessary for youth with undetectable levels of antibodies to the anti-HBs [14–16]. Multiple studies have reported the research of booster dose vaccine in youth [17,18], but small-sample studies are not universal, large-sample studies are needed.

The purpose of the present study was to investigate the positive conversion rates and positive maintenance rates of hepatitis B booster vaccine in youth through a large-sample study.

## Methods

### Study design and recipients

The present study recruited 37788 recipients aged ≤ 18 years who had received vaccinations at 0, 1, and 6 months after birth in Mianyang City, China. In addition, recipients who received insufficient interval doses or any other booster doses of hepatitis B vaccine were excluded. Hepatitis B surface antigen (HBsAg) of all involved recipients was negative before they received booster doses vaccination. Between June 2013 and October 2015, a recombinant Hepatitis B vaccine (*Saccharomyces cerevisiae*) with a dose of 10 μg (Hualan Biological Vaccine Company, Chengdu, China; 10 μg/0.5 ml) was used for all recipients at 0, 1, and 6 months, respectively. The biosynthesis of the HBsAg utilizes a recombinant plasmid containing S protein expressed in the host cell *S. cerevisiae* [19,20]. The strain producing the vaccine is a recombinant *S. cerevisiae* strain constructed by Merck, U.S.A. (strain number: 2150-2-3). The recipients were supplemented with baseline information including age, gender, nationality, family history of hepatitis B. Blood sample from each recipient was collected, and the serum was aseptically separated and stored at −20°C until testing. All recipients were divided into two groups based on their levels of anti-HBs before using the immune booster: anti-HBs negative group (anti-HBs(−)), the levels of anti-HBs < 10 mIU/ml; anti-HBs positive group (anti-HBs(+)), the levels of anti-HBs ≥ 10 mIU/ml. The present study was approved by the Institutional Review Board (IRB) of the West China Hospital, Sichuan University (approval number: No.2013(55)), and all the recipients signed written informed consent. The follow-up period was from June 2017 to January 2019.

### Laboratory testing

Serum levels of HBsAg and anti-HBs were analyzed by the ARCHITECT i2000SR analyzer (Abbott Laboratories; Chicago, IL, U.S.A.), and quantified by chemiluminescence microparticle immunoassay. The lower limit of detection of anti-HBs was 0.05 mIU/ml and samples with antibody levels above the range of the assay (250 mIU/ml) were diluted (1:500 or 1:1000) and re-test. The experiment process has strictly followed the instructions of the Abbott EIA AxSYM (Abbott, Abbott Park, IL, U.S.A.) and the operation manual of the instrument. Evaluation criteria for each indicator were the following: (1) recipient’s HBsAg serum concentration ≥ 0.05 IU/ml was defined as positive, otherwise it was negative; (2) recipient’s anti-HBs serum concentration ≥ 10 mIU/ml was defined as positive, otherwise it was negative.

### Statistical analysis

Categorical variables were expressed as the number and percentage (*n*, %). The chi-square test (χ^2^ test) was used to compare the positive rates of different characteristics of the research objects, and the multiple categorical variables with statistical differences were compared afterwards. The positive rate of anti-HBs was visualized at baseline, 1 year after vaccination, and 4 years after vaccination with grouped histogram. The Cochran–Armitage trend test was performed to determine the trend of anti-HBs positive rates. Statistical analysis was performed using the statistical software R (version 4.0.2), and *P*<0.05 was considered statistically significant.

## Results

### Characteristics of recipients

**Figure 1:**
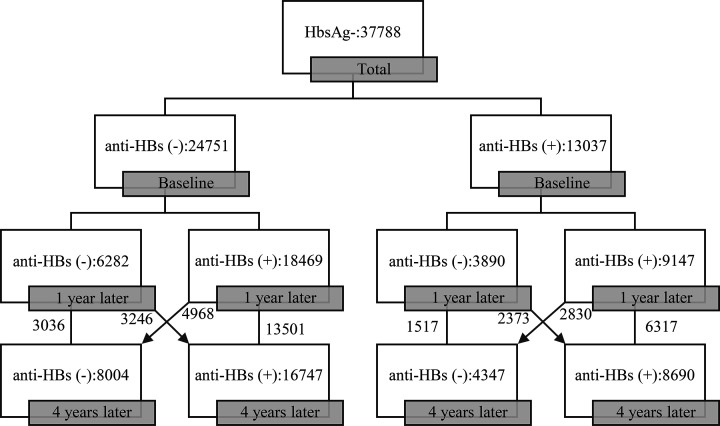
Overview of anti-HBs expression in all subjects

**Figure 2:**
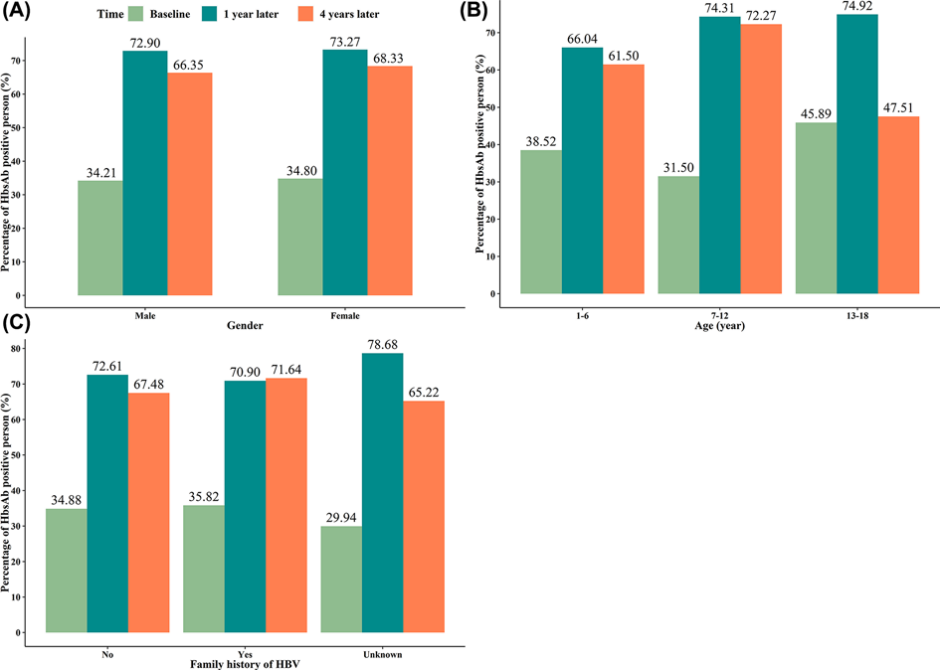
Visualizing the positive rate of anti-HBs at baseline, 1 year and 4 years after vaccination in all recipients (**A**) Based on gender; (**B**) based on age; (**C**) based on family history of HBV.

A total of 37788 recipients were involved in the present study, with mean age of 8.0 ± 2.8 years, and the ratio of male/female was 1.06/1. Of these, 34695 subjects (91.81%) had no family history of hepatitis B. Before hepatitis B booster vaccination, the positive rates of anti-HBs were 34.50%. At 1 and 4 years after hepatitis B booster vaccination, the positive rates of anti-HBs were 73.80% and 67.32%, respectively. The anti-HBs expression of the total recipients is shown in Figure 1. In addition, the positive conversion rates of HBsAg were 2.91 cases per 10000 people. The positive rates of anti-HBs based on gender, age (including 1–6 years, 7–12 years, and 13–18 years), and family history of hepatitis were shown in Figure 2 and Table 1.

**Table 1:** The expression of anti-HBs in all recipients at different time points

Characteristics	All recipients (*n*=37788)	Baseline		1 year later		4 years later	
		anti-HBs(+) (*n*=13037)	anti-HBs(−) (*n*=24751)	anti-HBs(+) (*n*=27616)	anti-HBs(−) (*n*=10172)	anti-HBs(+) (*n*=25437)	anti-HBs(−) (*n*=12351)
Gender, *n* (%)							
Male	19440 (51.44)	6651(34.21)	12789 (65.79)	14172 (72.90)	5268 (27.10)	12899 (66.35)	6541 (33.65)
Female	18348 (48.56)	6386 (34.80)	11962 (65.20)	13444 (73.27)	4904 (26.73)	12538 (68.33)	5810 (31.67)
Age (years), *n* (%)							
1–6	5971 (15.80)	2300 (38.52)	3671 (61.48)	3943 (66.04)	2028 (33.96)	3672 (61.50)	2299 (38.50)
7–12	26844 (71.04)	8455 (31.50)	18389 (68.50)	19947 (74.31)	6897 (25.69)	19401 (72.27)	7443 (27.73)
13–18	4973 (13.16)	2282 (45.89)	2691 (54.11)	3726 (74.92)	1247 (25.08)	2364 (47.54)	2609 (52.46)
Family history of HBV, *n* (%)							
No	34695 (91.81)	12103 (34.88)	22592 (65.12)	25193 (72.61)	9502 (27.39)	23411 (67.48)	11284 (32.52)
Yes	134 (0.35)	48 (35.82)	86 (64.18)	95 (70.90)	39 (29.10)	96 (71.64)	38 (28.36)
Unknown	2959 (7.83)	886 (29.94)	2073 (70.06)	2328 (78.68)	631 (21.32)	1930 (65.22)	1029 (34.78)

The relevant data of anti-HBs were expressed in numbers and percentages [*n* (%)]. ‘anti-HBs’, hepatitis B surface antibody; ‘anti-HBs(−)’, the level of anti-HBs < 10 mIU/ml; ‘anti-HBs(+)’, the level of anti-HBs ≥ 10 mIU/ml; ‘Baseline’, the time point before hepatitis B booster vaccination.

### The positive rates of anti-HBs in baseline anti-HBs(−) group

A total of 24751 subjects were anti-HBs negative before the hepatitis B booster vaccination. After receiving the hepatitis B vaccine, the positive conversion rates of anti-HBs were 74.62% (18469 cases) at 1 year after receiving booster vaccine, and 67.66% (16747 cases) at 4 years. There was statistically significant difference (χ^2^ = 291.43, *P*<0.001) in the positive conversion rates of anti-HBs at 1 and 4 years after vaccination. The negative conversion rates of anti-HBs within 1–4 years after vaccination were 20.07% (4968 cases), the positive conversion rates of anti-HBs within 1–4 years were 13.11% (3246 cases), and the positive maintenance rates of anti-HBs within 1–4 years were 54.55% (13501 cases).

**Figure 3:**
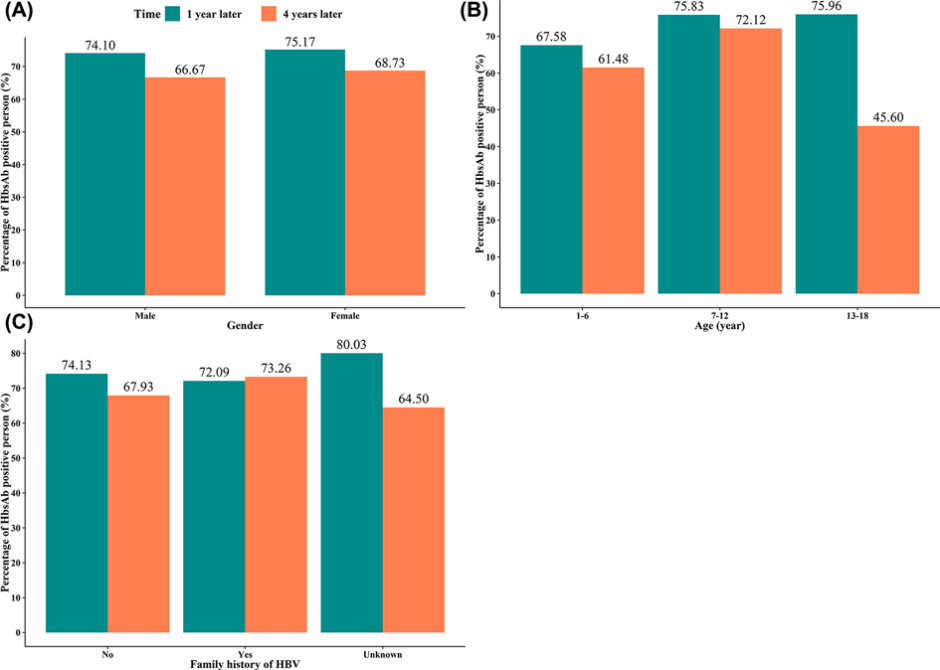
Visualizing the positive rate of anti-HBs in baseline anti-HBs(−) group (**A**) Based on gender; (**B**) based on age; (**C**) based on family history of HBV.

At 1 year after receiving the booster dose vaccine, statistical difference was observed in the positive rates of anti-HBs between different age groups (1–6 vs 7–12 vs 13–18: 67.58 vs 75.83 vs 75.96%) (*P*<0.001), especially, the positive rates of anti-HBs at the age group 1–6 years were the lowest. The positive rates of anti-HBs in different subjects with family history of HBV had statistical difference (*P*<0.001), but there was no statistical difference in the positive rates of those with family history of hepatitis B and those without family history (*P*>0.05). At 4 years after vaccination, the positive rates of anti-HBs between male and female had statistical difference (*P*=0.001), and the positive rates of male were lower (male vs female: 66.67 vs 68.76%). There was significant statistical difference in positive rates of anti-HBs in different age groups (1–6 vs 7–12 vs 13–18: 61.48 vs 72.12 vs 45.60%) (*P*<0.001), and the positive rates at the age 13–18 years were the lowest. Significant difference was observed in the positive rates of anti-HBs between different subjects with family history of HBV but there was no difference in the positive rates of those with family history of HBV and those without family history (*P*>0.05). Detailed statistical analysis information was shown in Table 2 and Figure 3.

**Table 2:** The positive rates of anti-HBs in baseline anti-HBs(−) group

Characteristics	1 year later			4 years later		
	anti-HBs(+) (*n*=18469)	anti-HBs(−) (*n*=6282)	*P*	anti-HBs(+) (*n*=16747)	anti-HBs(−) (*n*=8004)	*P*
Gender, *n* (%)			0.055			0.001
Male	9477 (74.10)	3312 (25.90)		8526 (66.67)	4263 (33.33)	
Female	8992 (75.17)	2970 (24.83)		8221 (68.73)	3741 (31.27)	
Age (years), *n* (%)			<0.001			<0.001
1–6	2481 (67.58)^1^	1190 (32.42)^1^		2257 (61.48)^1^	1414 (38.52)^1^	
7–12	13944 (75.83)^2^	4445 (24.17)^2^		13263 (72.12)^2^	5126 (27.88)^2^	
13–18	2044 (75.96)^2^	647 (24.04)^2^		1227 (45.60)^3^	1464 (54.40)^3^	
Family history of HBV, *n* (%)			<0.001			0.003
No	16748 (74.13)^1^	5844 (25.87)^1^		15347 (67.93)^1^	7245 (32.07)^1^	
Yes	62 (72.09)^1,2^	24 (27.91)^1,2^		63 (73.26)^1,2^	23 (26.74)^1,2^	
Unknown	1659 (80.03)^2^	414 (19.97)^2^		1337 (64.50)^2^	736 (35.50)^2^	

The relevant data of anti-HBs were expressed in numbers and percentages [*n*, (%)]. ‘anti-HBs’, hepatitis B surface antibody; ‘anti-HBs(−)’, the levels of anti-HBs < 10 mIU/ml; ‘anti-HBs(+)’, the levels of anti-HBs ≥ 10 mIU/ml; ‘Baseline’, the time point before hepatitis B booster vaccination. ‘1’, ‘2’, ‘3’ indicated comparison between groups, the same number (superscript) indicated that there was no statistical difference between the two rows, and the different numbers (superscript) indicated that the rows had statistical differences.

### The positive rates of anti-HBs in baseline anti-HBs(+) group

Before the hepatitis B booster vaccination, 13037 subjects were anti-HBs positive. After receiving the booster dose vaccine, the positive maintenance rates of anti-HBs were 70.16% (9147 cases) 1 year later, and 66.66% (8690 cases) 4 years later. There was significant difference (χ^2^ = 36.902, *P*<0.001) in the positive conversion rates of anti-HBs between 1 and 4 years. The negative conversion rates of anti-HBs within 1–4 years were 21.71% (2830 cases), the positive conversion rates of anti-HBs within 1–4 years were 18.20% (2373 cases), and the positive maintenance rates of anti-HBs within 1–4 years were 48.45% (6317 cases).

**Figure 4:**
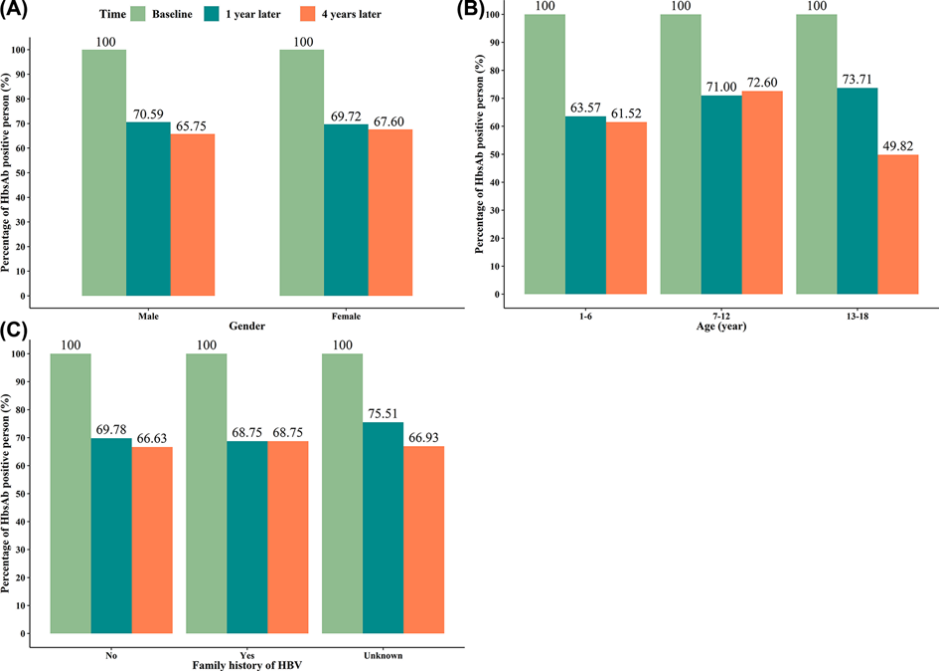
Visualizing the positive rate of anti-HBs in baseline anti-HBs(+) group (**A**) Based on gender; (**B**) based on age; (**C**) based on family history of HBV.

At 1 year after receiving the booster dose vaccine, there was significant difference (*P*<0.001) in the positive rates of anti-HBs in different age groups (1–6 vs 7–12 vs 13–18: 63.57 vs 71.00 vs 73.71%), especially the positive rates of the age group 13–18 years were the highest. There was no statistical difference in the positive rates of those with family history of hepatitis B and those without family history (*P*>0.05). Receiving the booster dose vaccine 4 years later, the positive rates of gender and age had statistical differences (all *P*<0.05). The positive rates of female and the age of 7–12 years was the highest. Strangely, the positive rates at age of 13–18 years were 49.82%, which was the lowest of all age groups (1–6 vs 7–12 vs 13–18: 61.52 vs 72.60 vs 49.82%). Detailed statistical analysis information was shown in Table 3 and Figure 4.

**Table 3:** The positive rates of anti-HBs in baseline anti-HBs(+) group

Characteristics	1 year later			4 years later		
	anti-HBs(+) (*n*=9147)	anti-HBs(−) (*n*=3890)	*P*	anti-HBs(+) (*n*=8690)	anti-HBs(−) (*n*=4347)	*P*
Gender, *n* (%)			0.283			0.026
Male	4695 (70.59)	1956 (29.41)		4373 (65.75)	2278 (34.25)	
Female	4452 (69.72)	1934 (30.28)		4317 (67.60)	2069 (32.40)	
Age (years), *n* (%)			<0.001			<0.001
1–6	1462 (63.57)^1^	838 (36.43)^1^		1415 (61.52)^1^	885 (38.48)^1^	
7–12	6003 (71.00)^2^	2452 (29.00)^2^		6138 (72.60)^2^	2317 (27.40)^2^	
13–18	1682 (73.71)^3^	600 (26.29)^3^		1137 (49.82)^3^	1145 (50.18)^3^	
Family history of HBV, *n* (%)			0.002			0.938
No	8445 (69.78)^1^	3658 (30.22)^1^		8064 (66.63)	4039 (33.37)	
Yes	33 (68.75)^1,2^	15 (31.25)^1,2^		33 (68.75)	15 (31.25)	
Unknown	669 (75.51)^2^	217 (24.49)^2^		593 (66.93)	293 (33.07)	

The relevant data of anti-HBs was expressed in numbers and percentages [*n*, (%)]. ‘1’, ‘2’, ‘3’ indicated comparison between groups, the same number indicated that there was no statistical difference between the two rows, and the different numbers indicated that the rows had statistical differences.

### Comparison of the positive rates between baseline anti-HBs(−) and anti-HBs(+) groups

Compared with baseline anti-HBs(+) group, the positive rates of anti-HBs were higher in baseline anti-HBs(−) group (χ^2^ = 86.014, *P*<0.001) at 1 year after receiving the booster vaccine. The positive rates of anti-HBs in baseline anti-HBs(−) group were slightly higher than anti-HBs(+) group (χ^2^ = 3.878, *P*=0.049) at 4 years after receiving the booster vaccine. The statistical results are shown in Table 4.

**Table 4:** Comparison of the positive rates between baseline anti-HBs(−) and anti-HBs(+) groups

	Baseline anti-HBs(−) group, *n* (%)	Baseline anti-HBs(+) group, *n* (%)	*P*
**1 year later**			<0.001
anti-HBs(+)	18469 (74.62)	9147 (70.16)	
anti-HBs(−)	6282 (25.38)	3890 (29.84)	
**4 years later**			0.049
anti-HBs(+)	16747 (67.66)	8690 (66.66)	
anti-HBs(−)	8004 (32.34)	4347 (33.34)	

The relevant data of anti-HBs were expressed in numbers and percentages [*n*, (%)].

### Cochran–Armitage trend test of anti-HBs positive rates after booster vaccination

The Cochran–Armitage trend test was performed to determine the trend between positive rates of anti-HBs with gender or each age group (Table 5). The results showed that there was an increasing trend of anti-HBs positive rates in all participants (Z = 91.480, *P*<0.001) after they received booster doses vaccination. In addition, an increasing trend of anti-HBs positive rates in males (Z = 64.163, *P*<0.001), females (Z = 65.249, *P*<0.001), age group of 1–6 years (Z = 25.255, *P*<0.001) and 7–12 years (Z = 96.182, *P*<0.001). However, no significant increasing trend of anti-HBs positive rates was observed in the age group of 13–18 years (*P*=0.098).

**Table 5:** The Cochran–Armitage trend test of anti-HBs positive rates after receiving booster vaccination

Populations, *n* (%)	Times	Subgroup		Z	*P*
		anti-HBs(+)	anti-HBs(−)		
Overall				91.480	<0.001
	Baseline	13037 (34.50)	24751 (65.50)		
	1 year	27616 (73.08)	10172 (26.92)		
	4 years	25437 (67.32)	12351 (32.68)		
Males				64.163	<0.001
	Baseline	6651 (34.21)	12789 (65.79)		
	1 year	14172 (72.90)	5268 (27.10)		
	4 years	12899 (66.35)	6541 (33.65)		
Females				65.249	<0.001
	Baseline	6386 (34.80)	11962 (65.20)		
	1 year	13444 (73.27)	4904 (26.73)		
	4 years	12538 (68.33)	5810 (31.67)		
1–6 years				25.255	<0.001
	Baseline	2300 (38.52)	3671 (61.48)		
	1 year	3943 (66.04)	2028 (33.96)		
	4 years	3672 (61.50)	2299 (38.50)		
7–12 years				96.182	<0.001
	Baseline	8455 (31.50)	18389 (68.50)		
	1 year	19947 (74.31)	6897 (25.69)		
	4 years	19401 (72.27)	7443 (27.73)		
13–18 years				1.657	0.098
	Baseline	2282 (45.89)	2691 (54.11)		
	1 year	3726 (74.92)	1247 (25.08)		
	4 years	2364 (47.54)	2609 (52.46)		

The relevant data of anti-HBs were expressed in numbers and percentages [*n*, (%)].

## Discussion

The present study involved 37788 recipients to evaluate the positive rates and positive maintenance rates of anti-HBs in youth with primary immunization after inoculation with a booster dose hepatitis B vaccine. We analyzed the positive rates of anti-HBs based on gender, age (including 1–6, 7–12, and 13–18 years), nationality, family history of HBV. The current study found that before hepatitis B booster dose vaccination, the positive maintenance rates of anti-HBs were 34.50% in primary immunization. After receiving the booster dose vaccine, the positive rates of anti-HBs were 73.80% at 1 year, and 67.32% at 4 years. The positive maintenance rates of anti-HBs were the lowest at the age of 13–18 years. The Cochran–Armitage trend test showed that there was an increasing trend of anti-HBs positive rates in all participants after receiving booster vaccination.

The results of anti-HBs(−) group indicated that the positive conversion rates were 74.62% at 1 year after receiving the booster doses vaccine, and 67.66% at 4 years. Compared with the previous study, the positive conversion rates were lower in our study at 1 year [21]. The reasons for this result may be the different vaccine doses and sample size from this study and the previous study. Previous studies have reported that vaccine dose significantly affects the positive rates of anti-HBs [22,23]. The study of Zhang et al*.* indicated that the anti-HBs seroconversion rate of the vaccine dose of 20-μg group was higher than that of 10-μg group (95.3 vs. 88.8%) [22]. In addition, some studies reported that due to factors such as age, gender, obesity, smoking, long-term drinking, and DRB1 and DQB1 HLA class II alleles, 5–30% of immune-competent individuals have not developed HBV serum protective, the levels of anti-HBs in their body are less than 10 mIU/ml [24,25]. Especially in population with low immune function, the percentage of undeveloped HBV serum protective is higher, and more immunogenic strategies should be adopted for these populations [2].

The persistence of anti-HBs and response to booster vaccination may be related to many factors, including the variety and dosage of vaccine used, the time interval between primary and booster vaccination, and the peak level of anti-HBs after full course of primary immunization [26,27]. Our results on the anti-HBs(+) group showed that the positive maintenance rates were 70.16% at 1 year after receiving the booster doses vaccine, and 66.66% at 4 years. The positive maintenance rates of antibody levels in the anti-HBs(+) group were lower compared with previous studies [17,27,28], especially in the age group of 13–18 years. Previous studies gave a possible explanation, that was, antibody titer decreased with age [29] and the titer was <10 mIU/ml at median 12.9 years [30]. Failure to detect antibody after vaccination did not mean that the vaccine is not protective. According to the report, while vaccine induced anti-HBs antibody, it also produced HBsAg-specific immune memory, which can provide continuous protection in the absence of antibody [31]. Furthermore, the sensitivity of antibody and antigen detection was also an important factor affecting the detection results. Several studies found that among the samples tested negative for HBsAg using conventional testing methods, 1–48% were tested positive using more sensitive HBsAg tests [32–34].

The present study explored the response of hepatitis B vaccine in youth based on gender, age, nationality, family history of HBV. The results of the present study may provide data support for future studies on hepatitis B vaccine. However, there were several limitations in our study. First, compared with non-obese people, obese individuals had a significantly lower antibody response to hepatitis B vaccine, and the risk of hepatitis B vaccine non-reactivity in obese people may increase with body mass index [35–37]. But the positive rates of anti-HBs based on body mass index were not analyzed in the present study. Future studies should be performed to explore the effect of obesity on the response to hepatitis B vaccine. Second, the varieties and doses of the hepatitis B vaccine given at the primary vaccination were also important factors that affected the antibody response, but they were not distinguished due to the obstruction of data collection.

## Conclusion

This is a large-sample study, which evaluated the positive rates of anti-HBs in youth at 1 and 4 years after receiving booster dose vaccine. The positive rates of anti-HBs decreased over time, especially the positive maintenance rates were the lowest at the age of 13–18 years. Longer follow-up studies are needed to assess the persistence of immunity in future researches. In addition, improving the success rates of vaccination and the sensitivity of antibody and antigen detection are attention-worthy.

## Data Availability

The data associated with the paper are available, and can be accessed through contacting first author (e-mail: xiazhusea2021@163.com).

## Abbreviations

anti-HBs: hepatitis B surface antibody
HBsAg: hepatitis B surface antigen
HBV: Hepatitis B virus
